# Similarity-Based Methods and Machine Learning Approaches for Target Prediction in Early Drug Discovery: Performance and Scope

**DOI:** 10.3390/ijms21103585

**Published:** 2020-05-19

**Authors:** Neann Mathai, Johannes Kirchmair

**Affiliations:** 1 Department of Chemistry and Computational Biology Unit (CBU), University of Bergen, N-5020 Bergen, Norway; neann.mathai@uib.no; 2 Department of Pharmaceutical Chemistry, Faculty of Life Sciences, University of Vienna, 1090 Vienna, Austria

**Keywords:** target prediction, molecular similarity, machine learning, random forest, molecular fingerprints, drug discovery

## Abstract

Computational methods for predicting the macromolecular targets of drugs and drug-like compounds have evolved as a key technology in drug discovery. However, the established validation protocols leave several key questions regarding the performance and scope of methods unaddressed. For example, prediction success rates are commonly reported as averages over all compounds of a test set and do not consider the structural relationship between the individual test compounds and the training instances. In order to obtain a better understanding of the value of ligand-based methods for target prediction, we benchmarked a similarity-based method and a random forest based machine learning approach (both employing 2D molecular fingerprints) under three testing scenarios: a standard testing scenario with external data, a standard time-split scenario, and a scenario that is designed to most closely resemble real-world conditions. In addition, we deconvoluted the results based on the distances of the individual test molecules from the training data. We found that, surprisingly, the similarity-based approach generally outperformed the machine learning approach in all testing scenarios, even in cases where queries were structurally clearly distinct from the instances in the training (or reference) data, and despite a much higher coverage of the known target space.

## 1. Introduction

Computational methods for predicting the macromolecular targets of small molecules have become increasingly relevant and popular in recent years due to (i) the shift from the “one-drug-one-target” paradigm to “polypharmacology” [1,2,3,4,5], (ii) the increasing availability of chemical and biological data [6,7,8] and (iii) advances in algorithms and hardware technology. Depending on the types of utilized data, in silico methods for target prediction may be categorized as ligand-based, structure-based, or hybrid methods [9,10,11,12]. Ligand-based methods range from straightforward similarity-based approaches [13,14,15,16,17,18,19,20,21] and linear regressions [22] to more complex machine learning (ML) models such as random forests [23,24,25], support vector machines [25,26,27], self-organizing maps [28], neural and deep neural networks [25,29,30,31,32,33,34], and network-based models [35,36,37,38]. They typically use large amounts of chemical information and measured bioactivity data [12] and, as a result, have a larger coverage of the target space when compared to structure-based methods, which rely on 3D structures of macromolecules. The third type of methods, hybrid approaches such as proteochemometrics and network-based approaches, utilize chemical, biological and structural information for target prediction.

Despite the abundance of in silico methods and models for target prediction that have been published in recent years, our understanding of their value and scope under (close to) real-world conditions remains limited [39]. In an ideal scenario, the performance of a model would be determined by large-scale prospective validation. However, the efforts and costs involved in running experiments on scales that can yield statistically meaningful conclusions are in general prohibitive. In consequence, to the best of our knowledge, the Similarity Ensemble Approach (SEA) method remains the only target prediction model that has undergone systematic experimental validation [40,41,42].

Most studies of new target prediction models are limited to retrospective validation [39]. Clearly, recent years have seen substantial progress in the implementation of more robust validation techniques of this kind, but one important aspect missed by most investigations is the relationship between the accuracy and reliability of predictions as a function of the distance between the compound of interest (query molecule) and the training data. In other words, reported validation studies often give a good idea of how well a model performs on the “average compound” originating from a defined dataset, but not so much about how trustworthy a prediction is for a particular compound of interest, which may or may not be structurally closely related to any of the instances in the training data. A further relevant point that is often not given the necessary consideration is target space coverage. Models trained and applied to targets for which a rich body of data is available will likely produce better performance statistics than models aiming to cover a wide target space. From the perspective of the end user, the most important question will be which method produces the most reliable predictions while covering the largest possible target space, and this question has been generally left unanswered.

This work aims to establish the value of two of the most common types of ligand-based methods for target prediction under conditions that closely resemble real-life applications: a straightforward similarity-based approach and a random forest-based ML approach, both employing Morgan2 fingerprints as representations of molecular structures. In particular, we investigate how the structural relationship between a query molecule and the molecules used for model training impact the reliability of predictions, and to what extent the individual approaches are able to cover the known target space.

## 2. Results and Discussion

### 2.1. Similarity-Based Method and Machine Learning Approach for Target Prediction

**Figure 1:**
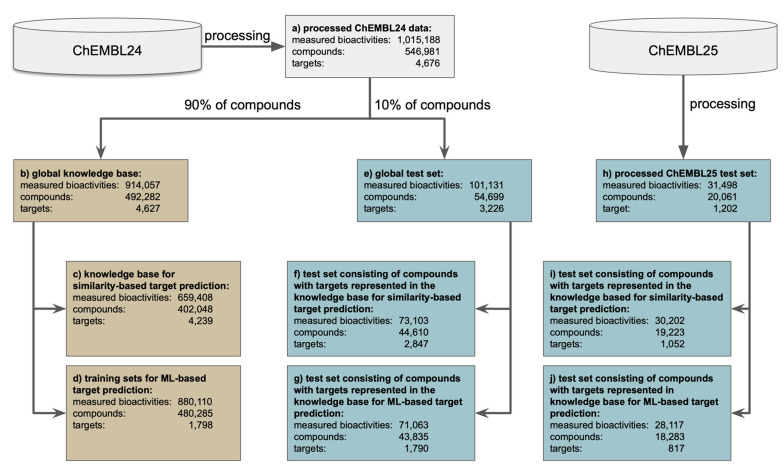
Creation of the knowledge base/training sets (brown) and test sets (blue) from version 24 (ChEMBL24) and version 25 (ChEMBL25) of the ChEMBL database. Measured bioactivities are counted as individual records (unique compound-protein pairs), compounds are counted as unique canonical SMILES of the preprocessed and standardized structures, and targets are counted as unique ChEMBL target IDs. The number of compounds reported in box d does not include the presumed inactive compounds used to create the individual training sets for the generation of the ML models.

Bioactivity data for model building and validation was extracted from the ChEMBL database [43] version 24. These data were curated and processed (see Methods section for details), resulting in a “processed dataset” consisting of 1,015,188 compound-protein pairs (546,981 unique compounds and 4676 unique targets; Figure 1a). Compound-protein pairs with an activity value less than or equal to 10,000 nM were marked as “active” (732,570 bioactivities) while those with activities greater than or equal to 20,000 nM were marked as “inactive” (282,618 bioactivities). Prior to any model development, the compounds in the processed dataset (Figure 1a) were randomly assigned to a “global knowledge base” (Figure 1b) or a “global test set” (Figure 1e) at a ratio of 90:10.

**Figure 2:**
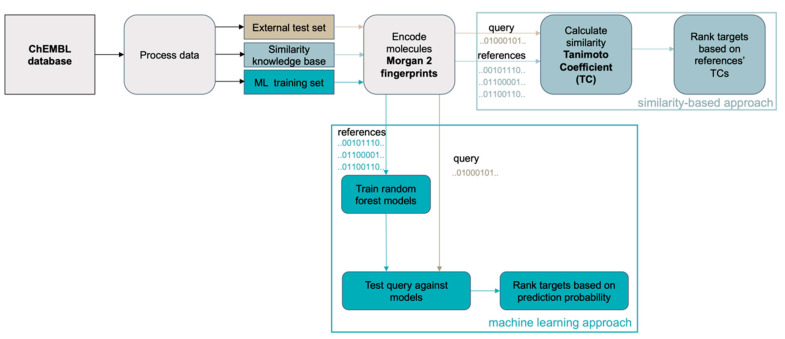
General workflow for the development and validation of the similarity-based method and the machine learning approach for target prediction.

The similarity-based approach uses the maximum pairwise similarities (Tanimoto coefficients (TC) derived from Morgan2 fingerprints; maxTCs) between a query molecule and the sets of ligands representing the 4239 individual proteins in the knowledge base (Figure 1c) to produce a rank-ordered list of potential targets (Figure 2). In cases where multiple proteins have the same maxTCs, the next highest TCs are considered until all proteins are ranked.

The ML approach decomposes the multi-label problem (i.e., a single query molecule may interact with many proteins) into a series of binary classification problems (i.e., a query molecule does or does not interact with a particular protein). This technique, known as binary relevance, is an intuitive and popular transformation [44] in target prediction [24,25,45]. Here, a query molecule is tested on all the target models individually and the models’ prediction probability of the active class (*p*-values) are then used to rank the potential targets for the query molecule (Figure 2). More specifically, random forest models were generated for each of the 1798 targets represented by a minimum of 25 ligands in the global knowledge base (Figure 1d). The individual models were trained on all active and all inactive compounds recorded for a target in the global knowledge base. Following a widely-applied approach in target prediction for expanding chemical space coverage [9,18,33,45,46], all training sets for ML for which the number of confirmed inactive compounds did not exceed the number of confirmed active compounds by a factor of 10 (this was the case for 1793 out of the 1798 targets) were supplemented with presumed inactive compounds (i.e., randomly chosen compounds from the global knowledge base which do not have any annotation for the particular target) to give a balance of 10:1. The hyperparameters of the individual random forest classifiers were optimized during a grid search within a cross-validation framework (see Method section for details).

### 2.2. Evaluation of the Scope and Performance of the Similarity-Based Method and Machine Learning Approach for Target Prediction

**Figure 3:**
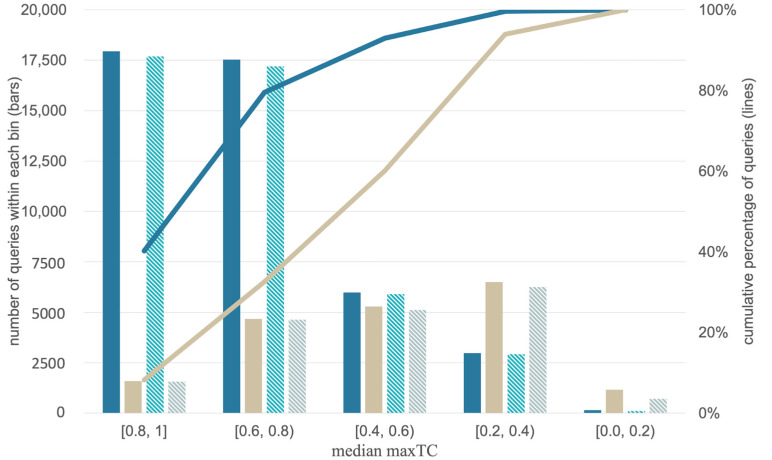
Distributions of the median maxTC values (quantifying the median molecular similarity of each query molecule and its nearest ligand of each of the query molecule’s annotated targets) for the queries from the ChEMBL24 database (blue) and ChEMBL25 database (grey). The results of the similarity-approach are marked without a pattern; the results for the ML approach are shown with a pattern. The bars represent the number of queries within a median maxTC bin. The distributions show that the ChEMBL24 test set is more similar to the data of the knowledge base than the ChEMBL25 test set (which is expected as the knowledge base is a subset of the ChEBML24 database). The lines report the cumulative percentage of queries with median maxTCs greater than or equal to the values covered by a bin. For the sake of clarity, the lines are only shown for the similarity-based approach as they are almost identical with the lines for the ML approach.

The scope and limitations of the individual approaches are evaluated under the following validation settings:

1. **Standard testing scenario with an external test set.** Under this scenario, the approaches are tested for their ability to predict the targets of a set of approximately 44,000 query molecules (Figure 1f,g) obtained by a single random split of the processed ChEMBL24 database prior to model development.
2. **Standard time-split validation scenario within the target space covered by the approach.** Under this scenario, the models are tested on the more than 18,000 molecules that have been newly introduced with version 25 of the ChEMBL database and have targets within the target space of the individual models (meaning that all compounds considered as queries have at least one known target that is covered by the approach’s knowledge base; Figure 1i,j). This test can give a sense of how model performance will change over time [39], with the increasing alienation of chemistry from that represented by the knowledge base (as observed in Figure 3).

1. **Close to real-world setting with an unbiased and comprehensive time-split dataset.** Under this scenario the methods were tested on the full set of bioactive compounds newly introduced with version 25 of the ChEMBL database (20,061 compounds; Figure 1h), regardless of whether or not any of the annotated targets is covered by the approach’s knowledge base. This scenario comes closest to real-world applications of target prediction methods, as there is a good chance that the targets of the new compounds (in particular those based on new chemistry) are novel, not represented by the training data, and hence missed by the in silico models.

Based on our experience in working with TCs derived from Morgan2 (and some related) fingerprints, we distinguish the following classes of queries:

- “High similarity queries”: These queries share a high degree of molecular similarity with the closest ligand (of the same target) in the knowledge base (TC greater than 0.66). Chemists will identify queries of this class as structurally closely related to the nearest ligand (of the respective target) in the knowledge base.
- “Medium similarity queries”: These queries share a moderate degree of molecular similarity with the closest ligand (of the same target) in the knowledge base (TC between 0.33 and 0.66). Chemists will typically find it challenging to identify obvious similarities between a query molecule of this class and the nearest ligand (of the respective target) in the knowledge base.
- “Low similarity queries”: These queries share a low degree of molecular similarity with the closest ligand in the knowledge base (TC lower than 0.33). Chemists will unlikely identify a query molecule of this class as structurally related to any of the ligands (of the respective target) in the knowledge base.

**Figure 4:**
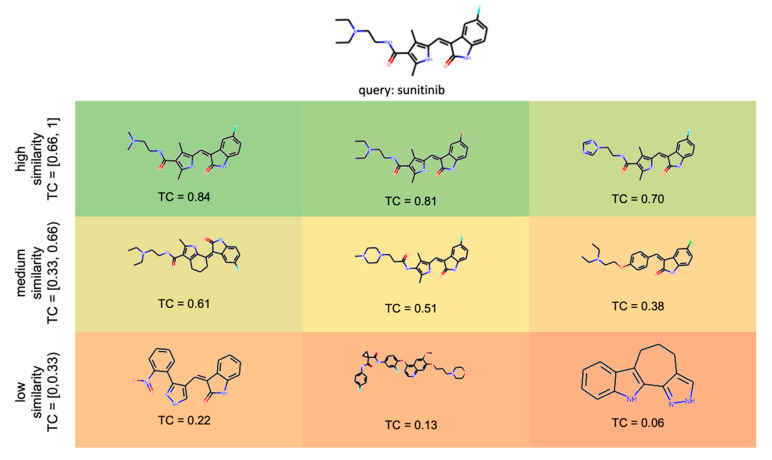
Query molecule (sunitinib) with examples of high, low, and medium similarity compounds from the ligand sets of its targets.

For the sake of clarity, the discussion of the methods’ performance focuses on these three categories (Figure 4 shows examples of different TCs of a query molecule and knowledge base ligands). A more fine-graded view is provided by the graphs and supplementary tables, which use a bin width for the TC of 0.2 for the disaggregation of the results. The trends observed at both levels of granularity are consistent.

#### 2.2.1. Evaluation of the Scope and Performance of the Similarity-Based Method

##### Performance in a Standard Testing Scenario with an External Test Set

**Figure 5:**
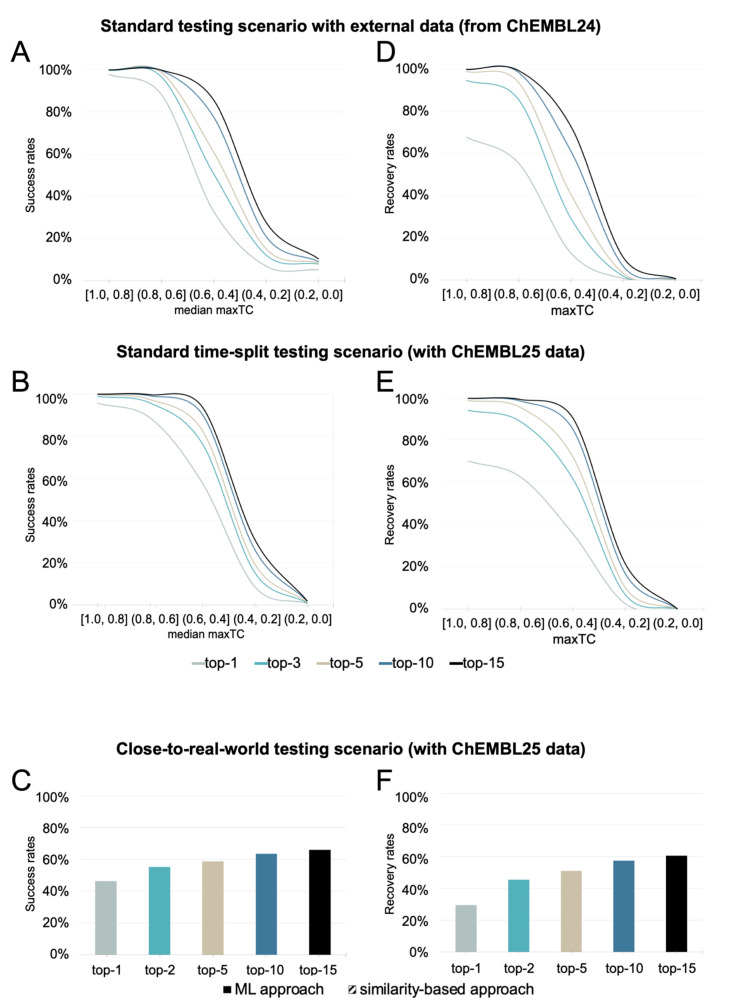
Success rates (**A**–**C**) and recovery rates (**D**–**F**) of the similarity-based approach under (**A**,**D**) the standard testing scenario with external data, (**B**,**E**) the standard time-split testing scenario, and (**C**,**F**) the close-to-real-world testing scenario. As expected, the performance under all testing scenarios drops as the queries become increasingly dissimilar to the data underlying the models. The data for these graphs are also provided in tabular format in the Supporting Information (Table S1–S4).

Overall, the similarity-based approach achieved high success rates, ranking at least one known target among the top-3, top-5, and top-15 positions in 86%, 88%, and 93% of all cases (Figure 5A). The success rates were found to be strongly linked with the distance between the query molecule and the nearest ligand (for that target) in the knowledge base. For 95% of all high similarity queries (as defined in the introductory section of the Results and Discussion section), the protein ranked at the top position was a known target. For medium similarity queries, however, the success rates were only 55%, 63%, and 82% when considering the top-3, top-5, and top-15 ranks, respectively. For low similarity queries, the success rates dropped to 10% (top-3), 12% (top-5), and 18% (top-15), respectively.

The similarity-based approach obtained good overall recovery rates (Figure 5D), with a strong correlation of performance and the distance between the query molecule and the compounds in the knowledge base observed here as well. The recovery rate is defined as the percentage of known bioactivities ranked among the top-*k* positions of the list of predicted targets. For high similarity queries, 92%, 97%, and 100% of the known targets were ranked among the top-3, top-5, and top-15 positions, respectively. In contrast, for medium similarity queries, the recovery rates were only 35%, 45%, and 70% when considering the top-3, top-5, and top-15 ranks, respectively. For low similarity queries, these success rates dropped to 1% (top-3), 1% (top-5), and 3% (top-15). 

##### Performance in a Standard Time-Split Testing Scenario

As expected, the success and recovery rates obtained for the similarity-based approach on the set of compounds newly introduced with version 25 of the ChEMBL database (“ChEMBL 25 test set”) were generally lower than for the ChEMBL24 test set (Figure 5B). The overall success rates among the top-3, top-5, and top-15 positions were 58%, 61%, and 69% (vs. 86%, 88%, and 93% obtained for the ChEMBL24 test set, see above). However, the success rates for queries represented by structurally related ligands in the knowledge base were comparable with those obtained for the ChEMBL24 test data: for high similarity queries, a known target was ranked at the top position in 93% of all cases (vs. 95% obtained for the ChEMBL24 test set). This is contrasted by the performance on medium similarity queries which had success rates of 70%, 76%, and 88% for the ChEMBL25 test set when considering the top-3, top-5, and top-15 ranks (vs. 55%, 63%, and 82% obtained for the ChEMBL24 test set). The success rates of the low similarity queries form the ChEMBL 25 test set dropped to 5%, 7%, and 13% for the top-3, top-5, and top-15 ranks (compared to 10%, 12%, and 18% for the ChEMBL 24 test set).

In accordance with the trends observed for the success rates, the recovery rates were also lower for queries from the new data in the ChEMBL25 database. Only 47%, 53%, and 63% of the known interactions were recovered among the top-3, top-5, and top-15 targets, as opposed to a recovery rate of 72%, 79%, and 87% obtained on the ChEMBL24 test set. Again, interactions with queries, which are more structurally related to the knowledge base, had higher recovery rates than those that were more distant (Figure 5E).

##### Performance in a Close-to-Real-World Testing Scenario

In the close-to-real-world testing scenario, an additional 1296 interactions (of 838 query molecules) not represented by the knowledge base were considered in the performance assessment. The 1296 interactions correspond to 4% of all interactions newly introduced with version 25 of the ChEMBL database. In consequence, the overall success rates for the top-3, top-5, and top-15 predictions decreased to 55%, 59%, and 66%, which represents a drop by 2 to 3 percentage points compared to the standard time-split scenario (Figure 5C). Likewise, the recovery rates for the top-3, top-5, and top-15 predictions dropped to 45%, 51%, and 61%, which is a decrease by 2 to 3 percentage points compared to the standard time-split scenario (Figure 5F).

##### Scope of the Similarity-Based Approach

The similarity-based approach has the widest scope of the approaches as any target with at least one known annotated ligand is represented in the knowledge base. The similarity-based approach covers a total of 4239 targets.

#### 2.2.2. Evaluation of the Scope and Performance of the Machine-Learning Approach

In the following subsections, the performance of the ML is discussed and directly compared to the performance of the similarity-based approach. For the sake of direct comparability, in the following discussion all statements on the performance of the similarity-based approach refer to its application to the reduced target space of the ML approach (1798 proteins) rather than the full target space covered by the similarity-based approach (4239 proteins). Note that, importantly, the target ranking performance of the similarity-based approach on the full target scope is almost identical to that on the reduced target scope, meaning that there is no noticeable drop in performance in the top-k success and recovery rates of the similarity-based approach (using identical absolute values for k) when applied to a target space of 4239 proteins instead of 1798, which is remarkable.

##### Performance in a Standard Testing Scenario with an External Test Set

**Figure 6:**
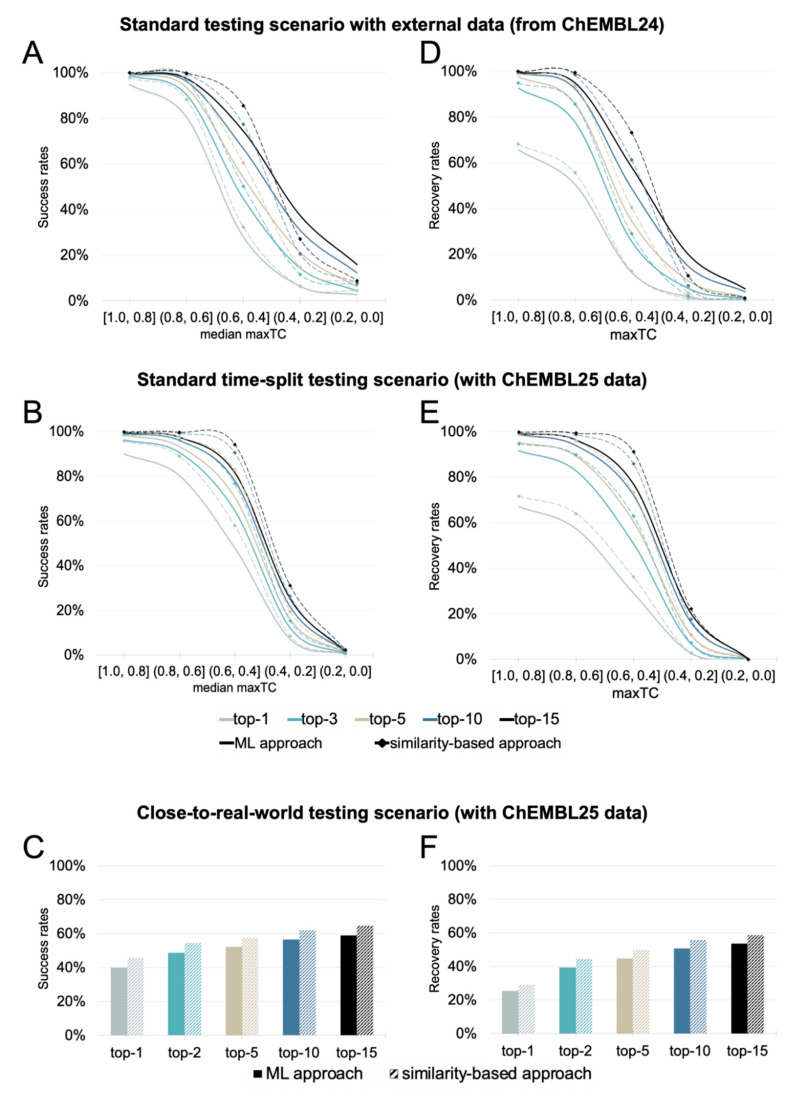
Success rates (**A**–**C**) and recovery rates (**D**–**F**) of the ML approach (solid lines and bars) and the similarity-based approach (dashed lines and bars; reduced target scope, identical with that of the ML approach) under the (**A**,**D**) standard testing scenario with external data, (**B**,**E**) the standard time-split testing scenario, and (**C**,**F**) the close-to-real-world testing scenario. In general, the similarity-based approach shows a tendency to outperform the ML approach. As expected, the performance under all testing scenarios drops as queries become more dissimilar from the training set/knowledge base. The data for these graphs are also provided in tabular format in the Supporting Information (Table S5–S12).

The ML approach achieved overall success rates of 82%, 86%, and 91% for the top-3, top-5, and top-15 positions, respectively (Figure 6A—solid lines), which is 1 to 3 percentage points lower than the success rates obtained by the similarity-based approach. The success rates were found to be strongly linked with the distance between the query molecule and the ligands in the knowledge base. For 90% of all high similarity queries, the known target was assigned the top rank (as compared to 95% for the similarity-based approach). For the median similarity queries, the success rates were only 49%, 57%, and 75% when considering the top-3, top-5, and top-15 ranks, respectively (which is 6 to 7 percentage points lower than the success rates of similarity-based approach). The success rates of the low similarity queries decreased to 10% (top-3), 15% (top-5), and 28% (top-15). Whereas the top-3 success rate was identical with that of the similarity-based approach, the top-5 and top-15 rates of the ML approach were 3 and 10 percentage points higher, respectively. This suggests that the ML approach may be able to predict the targets of low similarity queries better.

The recovery rates for the ML approach for high similarity queries were 88%, 94%, and 98% for the top-3, top-5, and top-15 positions, respectively (2 to 4 percentage points lower than the recovery rates of the similarity approach) (Figure 6D). For medium similarity queries, the recovery rates were only 30%, 39%, and 60% when considering the top-3, top-5, and top-15 ranks, respectively (5, 6 and 11 percentage points lower than the recovery rates of the similarity-based approach). The recovery rates for low similarity queries dropped to 3% (top-3), 5% (top-5), and 12% (top-15), which is still 2, 3, and 9 percentage points better than the values for the similarity-based approach.

##### Performance in a Standard Time-Split Testing Scenario

In line with the results obtained for the similarity-based approach, the success and recovery rates obtained for the standard time-split scenario were lower than for the standard testing scenario (Figure 6B). The overall top-3, top-5, and top-15 success rates were 53%, 57%, and 65%, respectively (vs. 82%, 86%, and 91% obtained for the ChEMBL24 test set; see above), which corresponds to 4, 3, and 1 percentage points below the time-split success rates of the similarity-based approach. For high similarity queries, a known target was ranked at the top position in 86% of all cases (which is 7 percentage points lower than the results obtained with the similarity-based approach). For medium similarity queries, the success rates were 59%, 65%, and 76% when considering the top-3, top-5, and top-15 ranks, respectively (vs. 49%, 57%, and 75% obtained for the ChEMBL24 test set). This corresponds to a drop by 11 to 13 percentage points over the similarity-based approach. For low similarity queries, the success rates were 4%, 7%, and 13% (nearly identical to the similarity-based approach in that scenario). 

The recovery rates were also lower for queries from the new data in the ChEMBL25 database. Only 44%, 50%, and 60% of all the known interactions were covered among the top-3, top-5, and top-15 predictions (Figure 6E), respectively (vs. 69%, 75%, and 84% for the ChEMBL24 test set). The trends observed for the recovery rates under the standard time-split scenario for the similarity and ML approach were the same as the success rates described above.

##### Performance in a Close-to-Real-World Testing Scenario

In the close-to-real-world testing scenario, an additional 3381 interactions (11% of the new interactions with version 25 of the ChEMBL database), which were not represented in the knowledge base, (from 1778 query molecules) were considered in the performance assessment. The overall success rates under this scenario were 49%, 52%, and 59% for the top-3, top-5, and top-15 predictions, respectively (Figure 6C). This represents a drop by 4 to 6 percentage points compared to the standard time-split scenario. The overall recovery rates for the top-3, top-5, and top-15 predictions dropped by 4 to 6 percentage points to 40%, 45%, and 54%, respectively (Figure 6F). In comparison to the similarity-based approach, the overall success rates under the close-to-real-world scenario were 6 percentage points lower for the top-3, top-5, and top-15 while the recovery rates were 5 percentage points lower.

##### Scope of the ML Approach

The 90:10 split of the processed ChEMBL24 data and the requirement for a minimum of 25 ligands per target for model building resulted in a reduced scope of the ML approach over the similarity-based approach. While the similarity-based approach covers a total of 4239 targets, the ML approach covers only 1798 targets (42% of the similarity-based approach’s target scope). As such, the ML approach did not cover 379 of the known targets of ChEMBL 24 queries, which resulted in the inability of the method to predict 2099 known interactions of 792 queries. 

The accuracy of the ML approach can be marginally increased by using larger training sets (Table 1), at the cost of target coverage. Models based on training sets consisting of a minimum of 50, 75 or 100 ligands (and ten times as many confirmed and/or presumed inactive compounds selected as described in the Methods section) would further reduce the number of proteins to 1296, 1066 and 899, respectively. The improvements in ranking performance are explained primarily by the reduction of the number of proteins represented by the approach, making ranking an easier task.

## 3. Methods

### 3.1. Data Preparation

Following a protocol closely related to that of Bosc et al. [24], the selection criteria listed below (italics indicate a ChEMBL data field and quotations indicate the value) were applied for the extraction of data from ChEMBL 24 [43]:

1. Assay covers a single protein or a protein complex (ChEMBL *confidence_score* is 7 or 9)
2. *data_validity_comment* is null OR “manually validated”
3. potential_duplicate is “0”
4. *standard_type* is “Kd”, “Potency”, “AC50”, “IC50”, “Ki”, or “EC50”
5. *activity_comment* is not “Inconclusive”, “inconclusive”, or “unspecified”
6. NOT (*standard_relation* is null AND *activity_comment* is not “Active” or “active”)
7. NOT (*standard_relation* “>”, “≥”, or “>>” AND *standard_value* less than 20,000)

This extraction procedure resulted in a dataset containing 1,482,972 bioactivity records (i.e., compound-protein pairs). Of these records, 2206 had standard_units of “μg mL^−1^” as opposed to “nM” and therefore the standard_value for these records were converted to nM using the topological information from *canonical_smiles* and the Descriptors.ExactMolWt function of RDKit (RDKit: Open-source cheminformatics; version 2019.03.2.0; http://www.rdkit.org). The molecules were then passed through the salt and element filter described in ref. [47], and the SMILES of the remaining compounds were converted with RDKit to non-isomeric SMILES. Duplicate compound-protein pairs, resulting from multiple bioactivity values recorded in the original data or from the removal of compound stereochemistry, were consolidated by calculating the median activity value as the representative activity value for the 1,179,102 unique bioactivity records. Compound-protein pairs with activity values less than or equal to 10,000 nM were labeled “active” (732,570 bioactivities) while those with activities greater than or equal to 20,000 nM were marked as “inactive” (282,618 bioactivities). Compound-protein pairs with activity values between 10,000 nM and 20,000 nM (163,914 bioactivities) were not considered for model building or validation and were discarded. The resulting dataset (“processed dataset”) consists of 1,015,188 compound-protein pairs, comprising 546,981 unique compounds and 4676 unique targets (Figure 1a) from which the knowledge base for the similarity-based approach (Figure 1c), the training sets for the ML approach (Figure 1b), and the testing sets (Figure 1e–g) were derived. Additionally, data from the next version of the ChEMBL database (version 25) was processed as described above and new bioactivity records were used for further test sets (Figure 1h–j)

### 3.2. Development of Target Prediction Models

#### 3.2.1. Similarity-Based Approach

The pairwise similarity of each compound of the test set (query molecule) and all compounds of the knowledge base for similarity-based target prediction was quantified based on TCs derived from Morgan fingerprints with a radius of 2 and a length of 2048 bits. Morgan2 fingerprints were selected because they are closely related to the extended connectivity fingerprints [48] with a diameter of 4 bonds (ECFP4), which have been widely applied in target prediction [16,17,25,49] and virtual screening [50] and have shown to perform favorably. For example, in tests of the target prediction methods Polypharmacology Browser (PPB2) [49] and MolTarPred [16,17], the ECFP4 fingerprints obtained the best performance among a collection of different molecular fingerprints.

The proteins were assigned ranks from 1 to 4239 (the total number of proteins represented by the knowledge base) according to the maximum pairwise TC of the query molecule and any of the ligands of that protein (maxTC). In cases where multiple proteins had the same maxTC, the ranking was refined based on the distance of the query molecule to the next nearest neighbor until non-ambiguous ranks could be assigned to all proteins.

#### 3.2.2. Machine Learning Approach for Target Prediction

Random forest binary classification models were built with scikit-learn (Scikit-learn: Machine Learning in Python; version 0.20.1; https://scikit-learn.org) [51] for all of the 1798 targets represented in the ML knowledge base by at least 25 ligands. The training set for each model is composed of the bioactivity records from the knowledge base and supplemented with presumed inactive compounds (selected randomly following the procedure described in the section “Generation of training and test sets” of Methods) to obtain a ratio of 1:10 compounds. The number of estimators (*n* estimators) and maximum depth of the estimators (max depth) of these models were optimized individually for each model during a grid search within a 10-fold cross-validation framework (Table 2). The best combination of parameters for each model (see the supplementary information), as measured by the average MCC score across the 10 folds, was then used to retrain the final model for each target using the complete training sets.

For a given query molecule, the proteins were then ranked by prediction probability of the active class (*p*-value). Proteins assigned identical *p*-values were ranked according to the iterative process described for the similarity-based approach.

## 4. Conclusions

This work aimed to determine the scope and limitations of two of the most commonly applied ligand-based methods for target prediction: a similarity-based approach, and a random forest-based machine learning approach, both employing Morgan2 fingerprints as molecular representations. By analyzing the performance of the approaches under three different scenarios and deconvoluting the results based on the distance of the test compounds (queries) from the training data (or knowledge base molecules), we obtained a robust and differentiated picture of the performance and reach of the approaches.

We have found that, in general, the similarity-based approach performed better than the ML approach, despite the similarity-based approach covering almost 2.5 times more proteins than the ML approach (4239 vs. 1798 proteins). Under the standard testing scenario with external data, the percentage of queries for which their target was recovered among the top-5 out of 1798 positions was 88% for the similarity-based approach and 85% for the ML approach (identical target space applied for the testing of both approaches). Under the time-split testing scenario, a drop in performance compared to the previous testing scenario was observed. Within a year’s time (i.e., the difference between ChEMBL24 and ChEMBL25), the top-5 success rate performance of all the approaches dropped by an average of 25% for the new chemical and target spaces explored during that year. This reduction in performance is expected, given the evolution of the chemical and target spaces over time, and is consistent with the drop performance observed for compounds with a comparable degree of (dis-) similarity with the training data in the standard testing scenario with external data. The results indicate that the single time-split scenario, which represents a snapshot of the evolution of research in small-molecule drug discovery, may not be essential for obtaining an understanding of the robustness of a method, provided that model performance is evaluated taking into account training-to-test set distances. The third scenario, which is closest to the real-life application of the method, looks into how the method would perform, taking into account that some of the targets of new molecules may not be covered by the model. Here, minor drops in model performance were observed.

It was surprising to find that overall the similarity-based approach outperformed the ML approach, particularly for the low-similarity queries, where it was hoped that the ML models would have generalized and been able to make more reliable predictions. It is probable that the ML approach may perhaps be improved with more and diverse data and achieve better generalization with more specific training protocols for individual targets.

## Figures and Tables

**Table 1:** Overall success and recovery rates of the ML approach when using individual target models with a different minimum number of active compounds.

	Success Rates				Recovery Rates			
Minimum number of actives (number of targets represented)	25 (1798)	50 (1296)	75 (1066)	100 (899)	25 (1798)	50 (1296)	75 (1066)	100 (899)
top-1	74.23% (32,539/43,835) ^1^	74.27% (31,897/42,946)	74.39% (31,369/42,170)	74.40% (30,754/41,336)	45.79% (32,539/71,063)	46.18% (31,897/69,066)	46.50% (31,369/67,457)	46.68% (30,754/65,880)
top-3	82.37% (36,107/43,835)	82.36% (35,370/42,946)	82.36% (34,730/42,170)	82.34% (34,035/41,336)	68.61% (48,756/71,063)	68.99% (47,649/69,066)	69.20% (46,681/67,457)	69.29% (45,651/65,880)
top-5	85.55% (37,503/43,835)	85.51% (36,725/42,946)	85.54% (36,072/42,170)	85.53% (35,353/41,336)	75.23% (53,460/71,063)	75.58% (52,197/69,066)	75.74% (51,095/67,457)	75.85% (49,970/65,880)
top-10	89.16% (39,083/43,835)	89.20% (38,308/42,946)	89.24% (37,634/42,170)	89.29% (36,909/41,336)	80.98% (57,545/71,063)	81.38% (56,205/69,066)	81.58% (55,034/67,457)	81.76% (53,863/65,880)
top-15	91.13% (39,946/43,835)	91.22% (39,175/42,946)	91.29% (38,496/42,170)	91.35% (37,759/41,336)	83.89% (59,617/71,063)	84.37% (58,270/69,066)	84.62% (57,083/67,457)	84.80% (55,866/65,880)

^1^ The percentage indicates the success and recovery rates, while the numbers in the brackets show how many queries (success rate) or bioactivities (recovery rate) within the TC interval had a hit.

**Table 2:** Hyperparameters explored in the grid search of each target classification model.

Hyperparameter	Values Explored
*n* estimators: number of trees	200, 500, 1000
max depth: maximum depth of tree	25, 45, 50, 75, 100

## Supplementary Materials

The following are available online at https://www.mdpi.com/1422-0067/21/10/3585/s1, Table S1. Success rates under the standard testing scenario with external data by the similarity approach, Table S2. Recovery rates under the standard testing scenario with external data by the similarity approach, Table S3. Success rates under the time-split and close-to-real-world testing scenarios by the similarity approach, Table S4. Recovery rates under the time-split and close-to-real-world testing scenarios with by the similarity approach, Table S5. Success rates under the standard testing scenario with external data by the similarity approach with a reduced target scope, Table S6. Recovery rates under the standard testing scenario with external data by the similarity approach with a reduced target scope, Table S7. Success rates under the time-split and close-to-real-world testing scenarios by the similarity approach with a reduced target scope, Table S8. Recovery rates under the time-split and close-to-real-world testing scenarios with by the similarity approach with a reduced target scope, Table S9. Success rates under the standard testing scenario with external data by the ML approach, Table S10. Recovery rates under the standard testing scenario with external data by the ML approach, Table S11. Success rates under the time-split and close-to-real-world testing scenarios by the ML approach, Table S12. Recovery rates under the time-split and close-to-real-world testing scenarios with by the ML approach.

## Abbreviations

ML	Machine learning
RF	Random forest
SMILES	Simplified molecular-input line-entry system
TC	Tanimoto coefficient
