# Supplementary material for: Similarity-Based Methods and Machine Learning Approaches for Target Prediction in Early Drug Discovery: Performance and Scope
Source: Int J Mol Sci. 2020 May 19;21(10):3585. doi: 10.3390/ijms21103585 (PMC7279241; doi:10.3390/ijms21103585)
Supplement: Supplementary file 1 [file ijms-21-03585-s001.zip › supplementary_tables.pdf]

International Journal of *Molecular Sciences*

MDPI

Supporting information for

Similarity-based methods and machine learning approaches for target prediction in early drug discovery: performance and scope

Neann Mathai[1] and Johannes *Kirchmair*[1,2,*]

[1] Department of Chemistry and Computational Biology Unit (CBU), University of Bergen, N-5020 Bergen, Norway

[2] Department of Pharmaceutical Chemistry, Faculty of Life Sciences, University of Vienna, 1090 Vienna, Austria

* Correspondence: johannes.kirchmair@univie.ac.at

The data of success and recovery rates presented in the graphs of the paper are reported below. The percentage indicates the success and recovery rates, while the numbers in the brackets show how many queries (success rate) or bioactivities (recovery rate) within the TC interval had a hit.

Similarity-based approach

Standard testing scenario with external data

Table S1. *Success rates under the standard testing scenario with external data by the similarity approach*

median maxTC	[0.8, 1]	[0.6, 0.8)	[0.4, 0.6)	[0.2, 0.4)	[0.0, 0.2)	overall
top-1	97.85% (17560/ 17946)	88.29% (15486/ 17539)	32.94% (1973/ 5989)	6.98% (208/ 2982)	5.19% (8/ 154)	78.98% (35235/ 44610)
top-3	99.74% (17899/ 17946)	96.61% (16945/ 17539)	50.63% (3032/ 5989)	12.17% (363/ 2982)	7.79% (12/ 154)	85.75% (38251/ 44610)
top-5	99.96% (17939/ 17946)	98.66% (17304/ 17539)	60.96% (3651/ 5989)	15.19% (453/ 2982)	8.44% (13/ 154)	88.23% (39360/ 44610)
top-10	99.98% (17943/ 17946)	99.70% (17486/ 17539)	77.63% (4649/ 5989)	21.09% (629/ 2982)	9.09% (14/ 154)	91.28% (40721/ 44610)
top-15	99.98% (17943/ 17946)	99.88% (17518/ 17539)	85.72% (5134/ 5989)	27.80% (829/ 2982)	10.39% (16/ 154)	92.89% (41440/ 44610)

Table S2. *Recovery rates under the standard testing scenario with external data by the similarity approach*

maxTC	[0.8, 1]	[0.6, 0.8)	[0.4, 0.6)	[0.2, 0.4)	[0.0, 0.2)	overall
top-1	67.82% (18368/ 27084)	55.34% (15480/ 27972)	12.65% (1341/ 10603)	0.66% (45/ 6767)	0.15% (1/ 677)	48.20% (35235/ 73103)
top-3	94.73% (25658/ 27084)	85.51% (23919/ 27972)	29.05% (3080/ 10603)	1.86% (126/ 6767)	0.15% (1/ 677)	72.20% (52784/ 73103)
top-5	98.91% (26788/ 27084)	93.59% (26179/ 27972)	40.48% (4292/ 10603)	3.16% (214/ 6767)	0.30% (2/ 677)	78.62% (57475/ 73103)
top-10	99.87% (27049/ 27084)	98.39% (27523/ 27972)	60.95% (6463/ 10603)	6.34% (429/ 6767)	0.44% (3/ 677)	84.08% (61467/ 73103)
top-15	99.96% (27074/ 27084)	99.32% (27782/ 27972)	72.76% (7715/ 10603)	10.64% (720/ 6767)	0.59% (4/ 677)	86.58% (63295/ 73103)

Standard time-split and close-to-real world testing scenarios

Table S3. *Success rates under the time-split and close-to-real-world testing scenarios by the similarity approach*

	time-split scenario						close-to-real-world scenario
**median maxTC**	**[0.8, 1]**	**[0.6, 0.8)**	**[0.4, 0.6)**	**[0.2, 0.4)**	**[0.0, 0.2)**	**overall**	
top-1	95.59% (1518/ 1588)	88.61% (4147/ 4680)	57.81% (3055/ 5285)	8.68% (565/ 6510)	0.69% (8/ 1160)	48.34% (9293/ 19223)	46.32% (9293/ 20061)
top-3	98.74% (1568/ 1588)	95.62% (4475/ 4680)	76.31% (4033/ 5285)	15.44% (1005/ 6510)	1.03% (12/ 1160)	57.71% (11093/ 19223)	55.30% (11093/ 20061)
top-5	99.75% (1584/ 1588)	97.37% (4557/ 4680)	82.65% (4368/ 5285)	19.43% (1265/ 6510)	1.38% (16/ 1160)	61.33% (11790/ 19223)	58.77% (11790/ 20061)
top-10	99.87% (1586/ 1588)	99.08% (4637/ 4680)	90.26% (4770/ 5285)	26.31% (1713/ 6510)	1.81% (21/ 1160)	66.21% (12727/ 19223)	63.44% (12727/ 20061)
top-15	99.87% (1586/ 1588)	99.62% (4662/ 4680)	93.72% (4953/ 5285)	31.08% (2023/ 6510)	1.98% (23/ 1160)	68.91% (13247/ 19223)	66.03% (13247/ 20061)

Table S4. *Recovery rates under the time-split and close-to-real-world testing scenarios with by the similarity approach*

	time-split scenario						close-to-real-world scenario
**maxTC**	**[0.8, 1]**	**[0.6, 0.8)**	**[0.4, 0.6)**	**[0.2, 0.4)**	**[0.0, 0.2)**	**overall**	
top-1	70.10% (1611/ 2298)	62.63% (4426/ 7067)	35.74% (2983/ 8346)	2.61% (273/ 10462)	0.00% (0/ 2029)	30.77% (9293/ 30202)	29.50% (9293/ 31498)
top-3	94.04% (2161/ 2298)	88.67% (6266/ 7067)	61.71% (5150/ 8346)	6.88% (720/ 10462)	0.00% (0/ 2029)	47.34% (14297/ 30202)	45.39% (14297/ 31498)
top-5	98.69% (2268/ 2298)	95.39% (6741/ 7067)	72.12% (6019/ 8346)	10.31% (1079/ 10462)	0.00% (0/ 2029)	53.33% (16107/ 30202)	51.14% (16107/ 31498)
top-10	99.70% (2291/ 2298)	98.40% (6954/ 7067)	85.15% (7107/ 8346)	16.93% (1771/ 10462)	0.00% (0/ 2029)	60.01% (18123/ 30202)	57.54% (18123/ 31498)
top-15	99.74% (2292/ 2298)	99.19% (7010/ 7067)	90.47% (7551/ 8346)	21.62% (2262/ 10462)	0.00% (0/ 2029)	63.29% (19115/ 30202)	60.69% (19115/ 31498)

Similarity-based approach - reduced scope

Standard testing scenario with external data

Table S5. *Success rates under the standard testing scenario with external data by the similarity approach with a reduced target scope*

median maxTC	[0.8, 1]	[0.6, 0.8)	[0.4, 0.6)	[0.2, 0.4)	[0.0, 0.2)	overall
top-1	97.81% (17307/ 17695)	88.21% (15177/ 17205)	32.28% (1904/ 5899)	6.43% (188/ 2923)	4.42% (5/ 113)	78.89% (34581/ 43835)
top-3	99.76% (17652/ 17695)	96.65% (16629/ 17205)	50.18% (2960/ 5899)	11.56% (338/ 2923)	7.08% (8/ 113)	85.75% (37587/ 43835)
top-5	99.96% (17688/ 17695)	98.70% (16981/ 17205)	60.54% (3571/ 5899)	14.64% (428/ 2923)	7.96% (9/ 113)	88.23% (38677/ 43835)
top-10	99.98% (17692/ 17695)	99.72% (17157/ 17205)	77.37% (4564/ 5899)	20.56% (601/ 2923)	7.96% (9/ 113)	91.30% (40023/ 43835)
top-15	99.98% (17692/ 17695)	99.88% (17185/ 17205)	85.61% (5050/ 5899)	27.10% (792/ 2923)	8.85% (10/ 113)	92.91% (40729/ 43835)

Table S6. *Recovery rates under the standard testing scenario with external data by the similarity approach with a reduced target scope*

maxTC	[0.8, 1]	[0.6, 0.8)	[0.4, 0.6)	[0.2, 0.4)	[0.0, 0.2)	overall
top-1	68.08% (18074/ 26550)	55.61% (15174/ 27287)	12.59% (1292/ 10265)	0.61% (40/ 6528)	0.23% (1/ 433)	48.66% (34581/ 71063)
top-3	94.92% (25201/ 26550)	85.72% (23391/ 27287)	29.00% (2977/ 10265)	1.81% (118/ 6528)	0.23% (1/ 433)	72.74% (51688/ 71063)
top-5	98.96% (26275/ 26550)	93.73% (25576/ 27287)	40.52% (4159/ 10265)	3.12% (204/ 6528)	0.46% (2/ 433)	79.11% (56216/ 71063)
top-10	99.87% (26516/ 26550)	98.48% (26872/ 27287)	61.27% (6289/ 10265)	6.31% (412/ 6528)	0.69% (3/ 433)	84.56% (60092/ 71063)
top-15	99.96% (26540/ 26550)	99.37% (27114/ 27287)	73.27% (7521/ 10265)	10.65% (695/ 6528)	0.92% (4/ 433)	87.07% (61874/ 71063)

Standard time-split and close-to-real world testing scenarios

Table S7. *Success rates under the time-split and close-to-real-world testing scenarios by the similarity approach with a reduced target scope*

	time-split scenario						close-to-real-world scenario
**median maxTC**	**[0.8, 1]**	**[0.6, 0.8)**	**[0.4, 0.6)**	**[0.2, 0.4)**	**[0.0, 0.2)**	**overall**	
top-1	95.84% (1496/ 1561)	88.91% (4122/ 4636)	58.04% (2971/ 5119)	8.51% (532/ 6251)	0.70% (5/ 716)	49.92% (9126/ 18283)	45.49% (9126/ 20061)
top-3	98.78% (1542/ 1561)	96.23% (4461/ 4636)	76.75% (3929/ 5119)	15.37% (961/ 6251)	0.84% (6/ 716)	59.61% (10899/ 18283)	54.33% (10899/ 20061)
top-5	99.74% (1557/ 1561)	97.58% (4524/ 4636)	82.77% (4237/ 5119)	19.53% (1221/ 6251)	1.54% (11/ 716)	63.17% (11550/ 18283)	57.57% (11550/ 20061)
top-10	99.87% (1559/ 1561)	99.29% (4603/ 4636)	90.56% (4636/ 5119)	26.25% (1641/ 6251)	1.96% (14/ 716)	68.11% (12453/ 18283)	62.08% (12453/ 20061)
top-15	99.87% (1559/ 1561)	99.72% (4623/ 4636)	94.30% (4827/ 5119)	31.04% (1940/ 6251)	2.23% (16/ 716)	70.91% (12965/ 18283)	64.63% (12965/ 20061)

Table S8*. Recovery rates under the time-split and close-to-real-world testing scenarios with by the similarity approach with a reduced target scope*

	time-split scenario						close-to-real-world scenario
**maxTC**	**[0.8, 1]**	**[0.6, 0.8)**	**[0.4, 0.6)**	**[0.2, 0.4)**	**[0.0, 0.2)**	**overall**	
top-1	71.53% (1583/ 2213)	63.97% (4368/ 6828)	36.22% (2904/ 8018)	2.75% (271/ 9860)	0.00% (0/ 1198)	32.46% (9126/ 28117)	28.97% (9126/ 31498)
top-3	94.62% (2094/ 2213)	89.81% (6132/ 6828)	62.91% (5044/ 8018)	7.22% (712/ 9860)	0.00% (0/ 1198)	49.73% (13982/ 28117)	44.39% (13982/ 31498)
top-5	98.78% (2186/ 2213)	95.88% (6547/ 6828)	73.15% (5865/ 8018)	10.87% (1072/ 9860)	0.00% (0/ 1198)	55.73% (15670/ 28117)	49.75% (15670/ 31498)
top-10	99.77% (2208/ 2213)	98.68% (6738/ 6828)	85.88% (6886/ 8018)	17.58% (1733/ 9860)	0.00% (0/ 1198)	62.47% (17565/ 28117)	55.77% (17565/ 31498)
top-15	99.77% (2208/ 2213)	99.36% (6784/ 6828)	91.16% (7309/ 8018)	22.28% (2197/ 9860)	0.00% (0/ 1198)	65.79% (18498/ 28117)	58.73% (18498/ 31498)

ML approach

Standard testing scenario with external data

Table S9. *Success rates under the standard testing scenario with external data by the ML approach*

median maxTC	[0.8, 1]	[0.6, 0.8)	[0.4, 0.6)	[0.2, 0.4)	[0.0, 0.2)	overall
top-1	94.93% (16797/ 17695)	80.73% (13889/ 17205)	28.16% (1661/ 5899)	6.47% (189/ 2923)	2.65% (3/ 113)	74.23% (32539/ 43835)
top-3	98.82% (17487/ 17695)	90.32% (15540/ 17205)	44.96% (2652/ 5899)	14.47% (423/ 2923)	4.42% (5/ 113)	82.37% (36107/ 43835)
top-5	99.38% (17586/ 17695)	93.57% (16098/ 17205)	54.08% (3190/ 5899)	21.28% (622/ 2923)	6.19% (7/ 113)	85.55% (37503/ 43835)
top-10	99.73% (17648/ 17695)	96.30% (16569/ 17205)	66.86% (3944/ 5899)	31.06% (908/ 2923)	12.39% (14/ 113)	89.16% (39083/ 43835)
top-15	99.84% (17667/ 17695)	97.49% (16773/ 17205)	74.47% (4393/ 5899)	37.46% (1095/ 2923)	15.93% (18/ 113)	91.13% (39946/ 43835)

***Table S10**. Recovery rates under the standard testing scenario with external data by the ML approach*

maxTC	[0.8, 1]	[0.6, 0.8)	[0.4, 0.6)	[0.2, 0.4)	[0.0, 0.2)	overall
top-1	65.62% (17423/ 26550)	50.49% (13777/ 27287)	12.06% (1238/ 10265)	1.53% (100/ 6528)	0.23% (1/ 433)	45.79% (32539/ 71063)
top-3	92.63% (24594/ 26550)	77.73% (21209/ 27287)	25.54% (2622/ 10265)	5.02% (328/ 6528)	0.69% (3/ 433)	68.61% (48756/ 71063)
top-5	97.65% (25927/ 26550)	85.82% (23418/ 27287)	34.60% (3552/ 10265)	8.53% (557/ 6528)	1.39% (6/ 433)	75.23% (53460/ 71063)
top-10	99.30% (26364/ 26550)	92.33% (25193/ 27287)	48.76% (5005/ 10265)	14.81% (967/ 6528)	3.70% (16/ 433)	80.98% (57545/ 71063)
top-15	99.66% (26461/ 26550)	94.86% (25885/ 27287)	57.99% (5953/ 10265)	19.87% (1297/ 6528)	4.85% (21/ 433)	83.89% (59617/ 71063)

Standard time-split and close-to-real world testing scenarios

Table S11. *Success rates under the time-split and close-to-real-world testing scenarios by the ML approach*

	time-split scenario						close-to-real-world scenario
**median maxTC**	**[0.8, 1]**	**[0.6, 0.8)**	**[0.4, 0.6)**	**[0.2, 0.4)**	**[0.0, 0.2)**	**overall**	
top-1	90.01% (1405/ 1561)	80.20% (3718/ 4636)	47.86% (2450/ 5119)	7.04% (440/ 6251)	0.42% (3/ 716)	43.84% (8016/ 18283)	39.96% (8016/ 20061)
top-3	96.28% (1503/ 1561)	90.55% (4198/ 4636)	64.43% (3298/ 5119)	12.01% (751/ 6251)	0.42% (3/ 716)	53.34% (9753/ 18283)	48.62% (9753/ 20061)
top-5	98.40% (1536/ 1561)	93.21% (4321/ 4636)	70.38% (3603/ 5119)	15.95% (997/ 6251)	0.70% (5/ 716)	57.22% (10462/ 18283)	52.15% (10462/ 20061)
top-10	99.30% (1550/ 1561)	96.05% (4453/ 4636)	77.93% (3989/ 5119)	21.52% (1345/ 6251)	1.12% (8/ 716)	62.05% (11345/ 18283)	56.55% (11345/ 20061)
top-15	99.62% (1555/ 1561)	97.43% (4517/ 4636)	81.46% (4170/ 5119)	25.05% (1566/ 6251)	1.26% (9/ 716)	64.63% (11817/ 18283)	58.91% (11817/ 20061)

Table S12. *Recovery rates under the time-split and close-to-real-world testing scenarios with by the ML approach*

	time-split scenario						close-to-real-world scenario
**maxTC**	**[0.8, 1]**	**[0.6, 0.8)**	**[0.4, 0.6)**	**[0.2, 0.4)**	**[0.0, 0.2)**	**overall**	
top-1	67.06% (1484/ 2213)	57.45% (3923/ 6828)	29.13% (2336/ 8018)	2.77% (273/ 9860)	0.00% (0/ 1198)	28.51% (8016/ 28117)	25.45% (8016/ 31498)
top-3	91.55% (2026/ 2213)	82.70% (5647/ 6828)	50.86% (4078/ 8018)	7.03% (693/ 9860)	0.00% (0/ 1198)	44.26% (12444/ 28117)	39.51% (12444/ 31498)
top-5	95.39% (2111/ 2213)	89.22% (6092/ 6828)	60.50% (4851/ 8018)	10.75% (1060/ 9860)	0.00% (0/ 1198)	50.20% (14114/ 28117)	44.81% (14114/ 31498)
top-10	98.73% (2185/ 2213)	93.85% (6408/ 6828)	72.11% (5782/ 8018)	16.33% (1610/ 9860)	0.08% (1/ 1198)	56.86% (15986/ 28117)	50.75% (15986/ 31498)
top-15	99.14% (2194/ 2213)	96.28% (6574/ 6828)	76.93% (6168/ 8018)	20.23% (1995/ 9860)	0.17% (2/ 1198)	60.22% (16933/ 28117)	53.76% (16933/ 31498)
